# The *Trichoderma reesei* Cry1 Protein Is a Member of the Cryptochrome/Photolyase Family with 6–4 Photoproduct Repair Activity

**DOI:** 10.1371/journal.pone.0100625

**Published:** 2014-06-25

**Authors:** Jesús Guzmán-Moreno, Alberto Flores-Martínez, Luis G. Brieba, Alfredo Herrera-Estrella

**Affiliations:** 1 Departamento de Biología, División de Ciencias Naturales y Exactas, Universidad de Guanajuato, Guanajuato, México; 2 Laboratorio Nacional de Genómica para la Biodiversidad, Cinvestav Irapuato, Irapuato, Guanajuato, México; The University of Hong Kong, Hong Kong

## Abstract

DNA-photolyases use UV-visible light to repair DNA damage caused by UV radiation. The two major types of DNA damage are cyclobutane pyrimidine dimers (CPD) and 6–4 photoproducts (6-4PP), which are repaired under illumination by CPD and 6–4 photolyases, respectively. Cryptochromes are proteins related to DNA photolyases with strongly reduced or lost DNA repair activity, and have been shown to function as blue-light photoreceptors and to play important roles in circadian rhythms in plants and animals. Both photolyases and cryptochromes belong to the cryptochrome/photolyase family, and are widely distributed in all organisms. Here we describe the characterization of *cry1*, a member of the cryptochrome/photolyase protein family of the filamentous fungus *Trichoderma reesei*. We determined that *cry1* transcript accumulates when the fungus is exposed to light, and that such accumulation depends on the photoreceptor Blr1 and is modulated by Envoy. Conidia of *cry1* mutants show decreased photorepair capacity of DNA damage caused by UV light. In contrast, strains over-expressing Cry1 show increased repair, as compared to the parental strain even in the dark. These observations suggest that Cry1 may be stimulating other systems involved in DNA repair, such as the nucleotide excision repair system. We show that Cry1, heterologously expressed and purified from *E. coli*, is capable of binding to undamaged and 6-4PP damaged DNA. Photorepair assays *in vitro* clearly show that Cry1 repairs 6-4PP, but not CPD and Dewar DNA lesions.

## Introduction

Fungi have the ability to respond to different environmental stimuli, including light, to regulate their growth, reproduction, and metabolism to adapt to the environment and survive adverse conditions. During evolution nearly all forms of life have been exposed to sun light, which given its optic properties can be used to produce thermodynamic work, and as a non-randomly structured system carries information. Radiation of short wavelength, corresponds to the ultraviolet (UV), and can initiate photochemical reactions. Among the molecules that can be affected by UV light, DNA must be highlighted, since the result of one of such reactions can be transmitted as a mutation to the next generation. Thus, light plays an ambiguous role in life, on one side all organisms depend on its energy and information, and on the other it is potentially harmful, and even deadly. Therefore, sunlight is a significant element for life, for which, during evolution, many mechanisms to resist its negative effects have been selected for [1].

*Trichoderma* photobiology has been studied for decades, leading to the discovery, first in *Trichoderma atroviride* and then in *Trichoderma reesei* of the blue light regulators (Blr1 and Blr2), which are essential for photoconidiation and gene expression regulated by blue light [2], [3], [4]. It has been postulated that Blr1 acts as the photoreceptor, in association with Blr2, in analogy with their *Neurospora crassa* counterparts WC-1 and WC-2 [1]. Recently, the influence of Blr1 and Blr2 on cellulase gene transcription has been shown in *T. reesei*, suggesting that these regulators act positively on this process [3]. In addition to the Blr proteins, in *Trichoderma* another photoreceptor Envoy (encoded by *env1*), a homolog of the *N. crassa* Vivid protein [5], [6], also plays an important role in light responses. Envoy is a negative regulator of the light input, switching off the expression of genes regulated by Blr1 and Blr2, and at least in *T. reesei* also modulates cellulase transcription in a light dependent manner [1], [3], [7]. Studies on the molecular basis of these light effects revealed that interconnections between the signaling pathways of light response, heterotrimeric G-proteins, the cAMP-pathway, sulfur metabolism, and oxidative stress are operative in *Trichoderma* [8], [9].

In a recent genome wide analysis of gene expression using pyrosequencing, 331 early light-regulated genes were identified, 70 of which appear to be blr-independent [1], [8]. These observations strongly suggest the existence of additional, functional light receptors. Indeed, the *T. atroviride and T. reesei* genomes encode potential photoreceptor proteins, such as phytochromes, cryptochromes and opsin-like proteins. A search for genes encoding potential blue-light photoreceptors in *T. reesei* revealed the presence of a CPD photolyase, a Cry-DASH-type cryptochrome, and a putative member of the cryptochrome/6-4 photolyase [1].

DNA-photolyases and cryptochromes are flavoproteins with high homology, which are involved in different light dependent biological processes, and are grouped within the cryptochrome/photolyase family of proteins. The cryptochrome/photolyase family consists of 55–70 kDa proteins that bind FAD, non-covalently, and an antenna chromophore of pterin (MTHF) or flavine type (8-HDF, FMN or FAD) in the photolyase homology region (PHR) [10], [11]. In addition, 6,7-dimethyl-8-ribityllumazine (DMRL) was recently found as an antenna chromophore in bacterial cryptochromes and photolyases [12], [13]. Photolyases repair DNA damage caused by UV light, through a process called photoreactivation. CPD photolyases repair cyclobutane pyrimidine dimers (CPDs) and 6–4 photolyases repair the pyrimidine (6–4) pyrimidone photoproducts [14], [15]. Cryptochromes are generally defined as photolyase-like proteins that have lost or have strongly reduced DNA repair activity and instead have gained signaling roles [11]. In organisms such as mammals and insects, cryptochromes are related to the metabolic and endocrine circadian clocks. Structurally, a C-terminal extension of variable length and with limited sequence similarity is present in both cryptochromes and 6–4 photolyases but is absent in CPD photolyases. Plants and animal Cry proteins have an additional C-terminal tail, cryptochrome C-terminus (CCT), in which the CCT sequence flanking the photolyase-like domain is involved in signaling, which can act either as a repressor or as a recognizing element for partner proteins [16]. Phylogenetic analysis of cryptochrome sequences indicates the existence of three main groups: plant cryptochromes, animal cryptochromes, and DASH-type cryptochromes [17]. Members of the animal Cry group are distributed from insects to vertebrates, and are involved in the control of circadian rhythms [18], [19], [20]. The Cry-DASH group comprises proteins that have weak single-strand DNA photorepair activity. Their signaling role, if any, is not clearly defined [21]. Plant Cry proteins were originally identified as blue light photoreceptors since they play important roles in blue-light mediated phototropic responses, development, and entrainment of the circadian clock [22], [23], [24]. Recently, a unique prokaryotic 6–4 photolyase, PhrB, has been reported in *Agrobacterium tumefaciens* that belongs to the group of iron-sulfur bacterial cryptochromes and photolyases (FeS-BCPs), and is distantly related to other cryptochrome/photolyase groups [13].

In fungi genes encoding proteins similar to cryptochrome/photolyases with probable functions in the perception of blue-UVA light have been found, however their function in most fungi is largely unknown [25], [26]. Members of the cryptochrome/photolyase family with functions, both as regulator cryptochrome-photolyase type and photorepair enzyme have been reported. Phr1, a protein with CPD photolyase activity that regulates its own expression, and modulates the expression of other light responsive genes in *T. atroviride*; and its closest orthologue from *A. nidulans*, CryA, shown to control sexual development in the fungus, are examples of the dual functions that these type of proteins might play [27], [28], [29]. In *Sclerotinia sclerotiorum cry1*, a DASH-type cryptochrome encoding gene is expressed in response to UV-A light and mutants in this gene show diminished sclerotial biomass, but this gene doesn't appear to play a relevant role in the life cycle of the fungus [30]. Similarly, in *N. crassa* expression levels of *cry*, a DASH-type encoding gene, are both light-induced and controlled by the circadian clock, a role in DNA repair has not been demonstrated for the corresponding protein, but purified Cry1 can bind single and double-stranded DNA and RNA [31]. The *Fusarium fujikuroi* Cry-DASH (*cryD*) gene expression is induced by light in the wild type strain but not in *wcoA* photoreceptor mutants, and participates in the regulation of secondary metabolism [32]. Yet another example is Phl1 from *Cercospora zeae*-*maydis*, which as a member of the cryptochrome/6-4 photolyase subfamily is involved in the repair of DNA damage by UV light, but is also involved in the regulation of the CPD photolyase, and participates in the development of the fungus and secondary metabolism [33].

Here we show that the *T. reesei* cryptochrome/6-4photolyase (Cry1) shares high sequence similarity and domain structure with other cryptochrome/6-4photolyase family members, including the FAD binding site and residues that potentially interact with (6–4) pyrimidine-pirimidone dimer (6-4PP). Transcript levels of *cry1* are induced by light in a *blr1* dependent manner and are repressed by *envoy*. Cry1 is capable of binding DNA in vitro as demonstrated by EMSA assays and shows 6–4 photolyase activity *in vivo* and *in vitro*.

## Materials and Methods

### Strains and culture conditions

The *T. reesei* QM9414 (ATCC 26921) parental strain and its derivatives, Δ*blr1* and Δ*env1* mutants [3] were used throughout this study. *T. reesei* was grown in plates with PDA medium (Difco) at 28°C. *E. coli* DH5α and TOP10F′ were used for plasmid DNA transformation, and *E. coli* SY2 (recA^−^, phr^−^, uvrA^−^) was used to express *cry1*. For isolation of protoplasts, cultures were grown in PDYC medium (24 g l^−1^ of potato dextrose broth, 2 g l^−1^ of yeast extract and 1.2 g l^−1^ of casein hydrolysate medium, all purchased from DIFCO). Transformed protoplasts were plated in PDA medium supplemented with hygromycin (100 µg ml^−1^) and overlayed with PDA soft agar containing 1% agar and hygromycin 100 µg ml^−1^ for transformant selection.

### Phylogenetic analysis of Cry1

Alignment of sequences was performed with ClustalW using BLOSUM 65 as a protein weight matrix without negative values and the phylogenetic and molecular evolutionary analyses were conducted using MEGA5 [34]. The sequences used for phylogenetic analysis with their respective accession numbers (gi) for NCBI protein database are presented below. **6–4 Photolyases**: *Trichoderma reesei* 340518659 or jgi: Trire2 77473 (**Cry1**), *Drosophila melanogaster* 22946921, *Xenopus laevis* 147906624, *Dunaliella salina* 61816948, *Danio rerio* 8698596, *Danaus plexippus* 133754344, *Colletotrichum orbiculare* 477526072, *Physcomitrella patens* 162694628, *Cercospora zeae-maydis* 170878123, *Oryza sativa Japonica* 51536259, *Coccomyxa subellipsoidea* 545363327, *Arabidopsis thaliana* 332642182, *Metarhizium robertsii* 374257344, *Trichoderma atroviride* 358394356, *Trichoderma virens* 358385715, *Phaeodactylum tricornutum 219118654*. **CPD Photolyases**: *Dunaliella salina* 118175518, *Oryza sativa Japonica* 70067250, *Chlamydomonas sp*. 469664987, *Chlamydomonas reinhardtii* 159469510, *Arabidopsis thaliana* 2984707, *Xenopus laevis* 147904876, *Trichoderma reesei* 340518124, *Trichoderma virens* 358381423, *Trichoderma atroviride* 358390515, *Fusarium oxysporum* 30315019, *Metarhizium anisopliae* 322707580, *Colletotrichum gloeosporioides* 530478867, *Beauveria bassiana* 400602074, *Neurospora crassa* 553140027, *Magnaporthe oryzae* 389623797. **Cry-DASH**: *Arabidopsis thaliana* 332005986, *Xenopus laevis* 147902555, *Vibrio cholerae* 550385215, *Colletotrichum orbiculare* 477535376, *Verticillium dahliae* 346970183, *Danio rerio* 68534519, *Neurospora crassa* 553139799, *Sclerotinia borealis* 563297898, *Synechococcus sp* 493502164, *Trichoderma atroviride* 358394234, *Trichoderma reesei* 340519796, *Trichoderma virens* 358384981, *Magnaporthe oryzae* 440472686. **Crys**: *Drosophila melanogaster* 3983154, *Danaus plexippus* 357603254, *Xenopus laevis* 147901075, *Danio rerio* 390979651, *Gallus gallus* 110626125, *Rattus norvegicus* 33333729, *Gallus gallus* 19772572. **FeS-BCPs**: *Agrobacterium tumefaciens* 480311930, *Rhodobacter sphaeroides* 375332625.

### Generation of *T. reesei cry1* mutant an overexpressing strains

In order to obtain a *cry1::hph* mutant, the Double Joint PCR method was performed as previously described by Yu *et al.* [35]. The primers used to construct the replacement cassette of the *cry1* gene are described in Table 1. Gene sequence was obtained from the *T. reesei*, v2.0 genome database http://genome.jgi-sf.org/Trire2/Trire2.home. First, 100 ng of genomic DNA template were used for PCR amplification of the 5′ and 3′ flanking regions of cry1 with primers cry1-1, and cry1-2, and cry1-3 and cry1-4, respectively. In a parallel PCR reaction, 1 ng pUE08 plasmid was used for amplification of the hygromycin B phosphotransferase (*hph*) marker [36]. In a third PCR reaction, the replacement cassette *cry1::hph* was amplified using 1 µl of the purified products obtained in the second PCR reaction as template, and cry1-5 and cry1-6 primers. The identity of the replacement cassette was confirmed by restriction pattern and purified using QIAquick Spin Columns (Qiagen). 20 µg of the purified product was used for PEG-mediated protoplast transformation of the QM9414 strain. For over-expression of *cry1* we introduced the open reading frame of *cry1* into the *EcoR*I-site of the vector pUE08 [36], resulting in pOE*cry1*, which expresses *cry1* under the control of the *T. reesei pki* promoter and *TtrpC* terminator of *A. nidulans*. pOE*cry1* was used to transform *T. reesei* QM9414 as previously described by Baek and Kenerley [37], except that mycelium was treated with 120 mg ml^−1^ of enzymatic extract of *Trichoderma harzianum* (Sigma). After three rounds of monosporic culture, fungal DNA was isolated from putative Δ*cry1* mutants and OE*cry1* transformed strains using standard protocols. Gene replacement events were initially identified by PCR, and confirmed by Southern-blotting.

**Table 1:** Primers used in this work.

Primer	5'- 3' Sequence
Cry1-1	GCATGAGCCGAGTTTGTCTTG
Cry1-2	GGACGACTAAACCAAAATAGGCATTCATTGTGCGCTGGAAGTCTTGTATGTAAGG
Cry1-3	GCACTCGTCCGAGGGCAAAGGAATAGCAGAGTATAACATGTAATTAGAGAACACT
Cry1-4	GCTGCCGACAACATCAACTTC
Cry1-5	GTCTCGGTGGGATATGAGTAG
Cry1-6	AGTTGCCCGAGGATGTTGC
ORFCry-f	TCTAGATCC AGC GCA ATG ACC AAG C
ORFCry-r	ATCGATCTCTGAAGCCAGGCTATTCTTG
pColdF	CAT*ATG* ACCAAGCCGCGTGTGATTTAC
pColdR	TCTAGA *CTA*TTCTTGTGTCTTCTGCTTC

### Northern and Southern blot analysis

Genomic DNA was isolated following the procedure described by Raeder and Broda [38]. Total RNA was isolated according to the protocol described by Jones *et al* [39]. Southern and Northern blotting were performed using Hybond-N^+^ membranes (Amersham) hybridized with probes labeled by random priming with [α-^32^P]dCTP and processed by standard procedures [40]. Southern Blot analysis of the *cry1* mutants was carried out by restriction of 10 µg of the Δ*cry1* mutant genomic DNA with the restriction enzyme *Sma*I, which cuts in the middle of the *cry1* gene. Genomic DNA was also digested with the restriction enzyme *Nco*I, which cuts in the middle of the *hph* resistance cassette. The fragments of genomic DNA were separated by electrophoresis in a 1% agarose gel and transferred onto a nitrocellulose membrane (Amersham) which was hybridized with a DNA fragment containing sequences of the promoter, ORF or terminator of *cry1*, as indicated.

Southern Blot analysis of OE*cry1* mutants was carried out by restriction of 10 µg of genomic DNA with *EcoRI*, which releases a 1.9 kb fragment corresponding to the *cry1* gene contained in the integrated construct. Genomic DNA was also digested with the restriction enzyme *Sal*I that cuts in the *cry1* gene. Restriction fragments were separated by electrophoresis in 1% agarose gel, transferred onto a nitrocellulose membrane (Amersham) and hybridized with a DNA fragment containing the ORF of *cry1*.

### Blue light photoinduction

Spores of the strains used for photoinduction assay were plated in PDA and incubated at 28°C during 48 h. For gene expression analysis plugs of mycelia (0.5 cm diameter) were obtained from the colony edges and placed on the center of PDA plates with cellophane overlay and incubated during 36 h in the dark before light exposure. Colonies were subjected to a 5 min pulse of blue light (450 nm; 1,200 µmol m^−2^) in a light emitting diode (LED) chamber (Percival) at 28°C and plates taken back to darkness. Samples of mycelium were collected at 15, 30, 60, and 120 min, frozen immediately in liquid nitrogen, and stored until used for RNA extraction. Gene expression was also analyzed upon continuous exposure of the colonies for 5, 15, and 30 min to blue light (3.6 µmol m^−2^ s^−1^), and samples collected after exposure to light, frozen in liquid nitrogen, and stored for later analysis. All manipulations of the mycelium were carried out in a dark room using red light of security.

### Cloning of *cry1* and protein purification from *E. coli*

Total RNA was treated with RNAse-free DNAse I (Invitrogen) and cDNA synthesis was performed using 1 µg of RNA and superscript RT II (Invitrogen) following the manufacturer recommendations. The cDNA sequence encoding *cry1* was PCR amplified using primers, pColdF and pColdR, designed to add an *NdeI* site proximal to the start codon and an *XbaI* site after the stop codon. The product was digested and cloned into the pColdI vector (Takara) and the resulting N-terminally six-His-tagged expression construct (pCold-cry1) was analyzed by sequencing to confirm that the *cry1* gene was in correct sense and used to transform electrocompetent *E. coli* SY2 strain. Transformed SY2 cells were grown at 37°C until 0.4 OD_600_, cooled in ice 30 min and induced at 15°C for 24 h using 0.1 mM IPTG. The soluble protein fraction was purified by chromatography using a HIS-Trap FF column (GE Healthcare Life Sciences) according to the manufacturer's recommendations. The protein was eluted and dialyzed in phosphate buffer (sodium phosphate monobasic 50 mM, pH 8, NaCl 50 mM, 1 mM PMSF, TCEP 1 mM and 1 mM EDTA) for 12 h, and used in a second round of purification in a monoQ anion exchange column (BioRad), eluting with a 50 mM to 1.5 M NaCl gradient. This protein was used for EMSA assays and *in vitro* photorepair assays.

### Homology model

An homology model was build with MOE using as a template the crystal structure of a photolyase from *Drosophila melanogaster* in complex with 6–4 thymine dimer (PDB ID: 3CVU). Both proteins share 39% amino acid identity until residue 555 of Cry1, the non-conserved C-terminal 73 amino acids of Cry1 were not included in the model. The final model was selected from 25 initial models constructed with the CHARMM27 force fields and energy minimized.

### Electrophoretic mobility shift and *in vitro* photorepair assays

Gel shift binding experiments and DNA photorepair assays were performed using as substrate a double stranded DNA probe of 49 bp containing a single UV photoproduct (either CPD, 6–4 photoproduct or a DEWAR isomer) at a *Mse*I restriction site (prepared as described in Hitomi *et al* [41]. Dewar isomer is a photoproduct derived from the irradiation of 6–4 photoproducts at 313 nm. The substrate sequence is as follows: d(AGCTACCATGCCTGCACGAATTAAGCAATTCGTAATCATGGTCATAGCT), and the thymine dimer of the damaged strand is underlined. The complementary oligonucleotide was labeled with [γ-^32^P] ATP by T4 polynucleotide kinase and annealed with the damaged strand by heating at 95°C for 5 min and cooling to room temperature for 2–3 h. The labeled duplex DNA, containing a single photoproduct, was used as substrate for both assays. For the EMSA assay 0, 4.23, 8.45, 12.68 and 25.35 µM of purified recombinant proteins were incubated with 1 nM of ^32^P-labelled DNA substrate and subsequently analyzed by electrophoresis on a 10% non-denaturing acrylamide gel. The *in vitro* repair assay was carried out using 7 µM of purified Cry1 protein mixed with 10 nM of ^32^P-labelled DNA substrate, the reaction mixtures containing the enzymes were illuminated for 20 min using daylight fluorescent lamps in 100 mM Tris-HCl pH 8.0, 1 mM DTT. The DNA substrate was subjected to *Mse*I digestion and finally analyzed by electrophoresis on a denaturating acrylamide gel.

### Photoreactivation assay *in vivo*

Photoreactivation assays (drops) were conducted in the following manner, fresh conidia were collected and counted in a Neubauer Chamber and 200 conidia from different strains to test, in 5 µl sterile water, placed on PDA medium, allowed to dry and irradiated with UV-C light using a Stratalinker UV 2400 (Stratagene) at a 350 J m^−2^ dose incubating them later at 28°C for 24 h under conditions of constant light (for photoreactivation) or in the dark (control). As control the same strains (non-irradiated) were grown under the same conditions. Finally they were observed under a microscope to evaluate germination of conidia, as a measure of photoreactivation. The experiments were carried out in duplicate with three technical replicates for each condition.

Photoreactivation assays (survival) 200 fresh conidia were spread on PDA plates with Triton-X100 0.5% (to restrict growth). The plates were let to dry, and irradiated with UV-C at a dose of 350 J m^−2^ and subsequently incubated at 28°C for 48 h under constant light conditions (for photoreactivation) or in the dark (control). As control non-irradiated strains were grown under the same conditions. The colonies on each plate were counted and the number plotted as percent survival relative to non-UV-treated conidia. The experiments were carried out in duplicate with three technical replicates for each condition.

## Results

### The *T. reesei* Cry1 belongs to the cryptochrome/6-4 photolyase family

**Figure 1:**
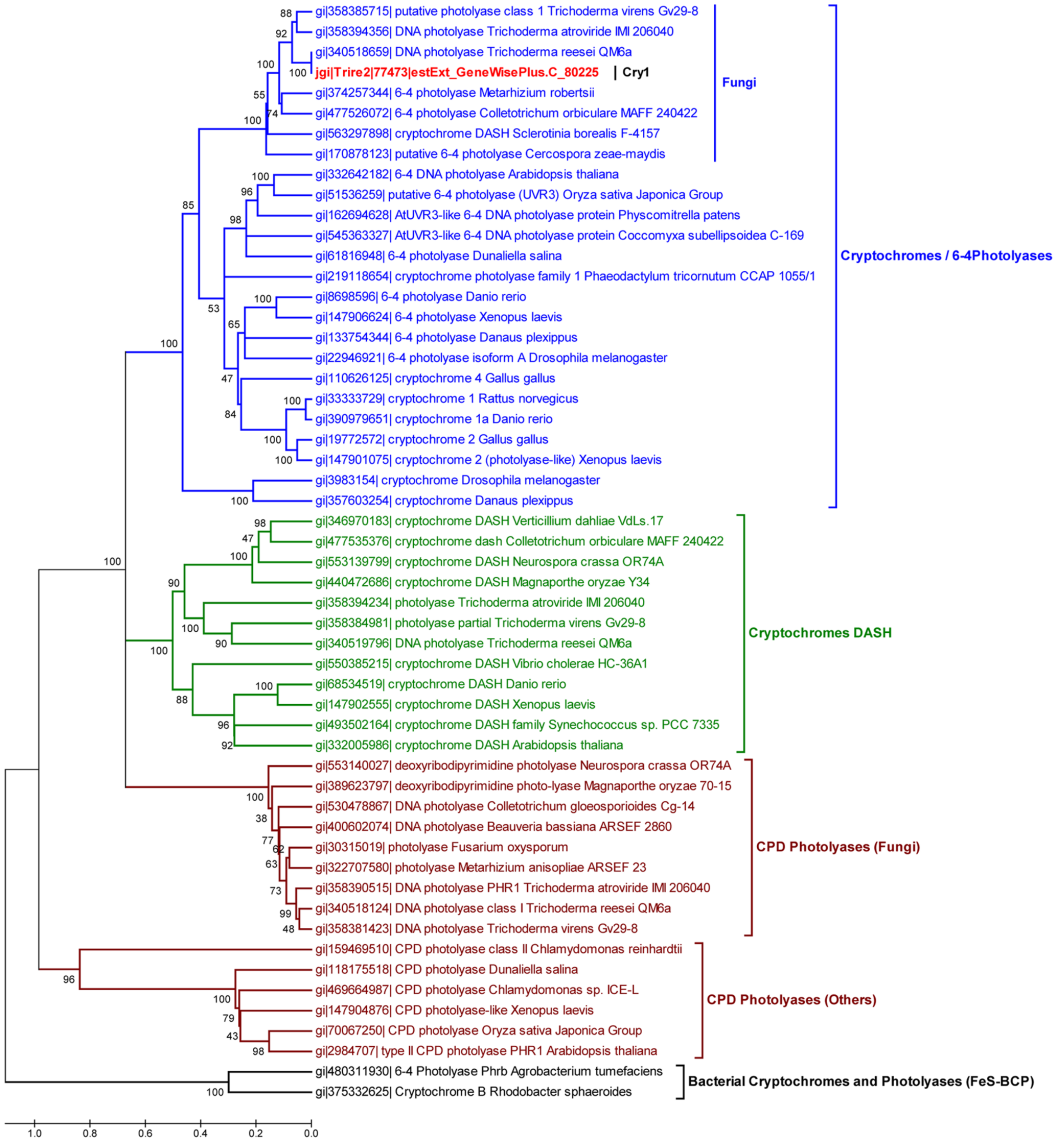
Cry1 is closely related to the cryptochrome/6-4 photolyase family. Analysis of *cry1* based on multiple sequence alignments with some members of the cryptochrome/photolyase family. In blue cryptochrome/6-4 photolyase family, in green DASH cryptochromes, in red CPD photolyases and black bacterial cryptochromes and photolyases, is shown and the NCBI sequence identifier (gi) for each protein. The evolutionary history was inferred using the Minimum Evolution method. The optimal tree with the sum of branch length  = 15.50943863 is shown. The percentage of replicate trees in which the associated taxa clustered together in the bootstrap test (1000 replicates) is shown next to the branches. The tree is drawn to scale, with branch lengths in the same units as those of the evolutionary distances used to infer the phylogenetic tree. The evolutionary distances were computed using the Poisson correction method and are in the units of the number of amino acid substitutions per site. The ME tree was searched using the Close-Neighbor-Interchange (CNI) algorithm at a search level of 1. The Neighbor-joining algorithm was used to generate the initial tree. The analysis involved 54 amino acid sequences. All positions containing gaps and missing data were eliminated. There were a total of 300 positions in the final dataset. Evolutionary analyses were conducted in MEGA5 [34].

**Figure 2:**
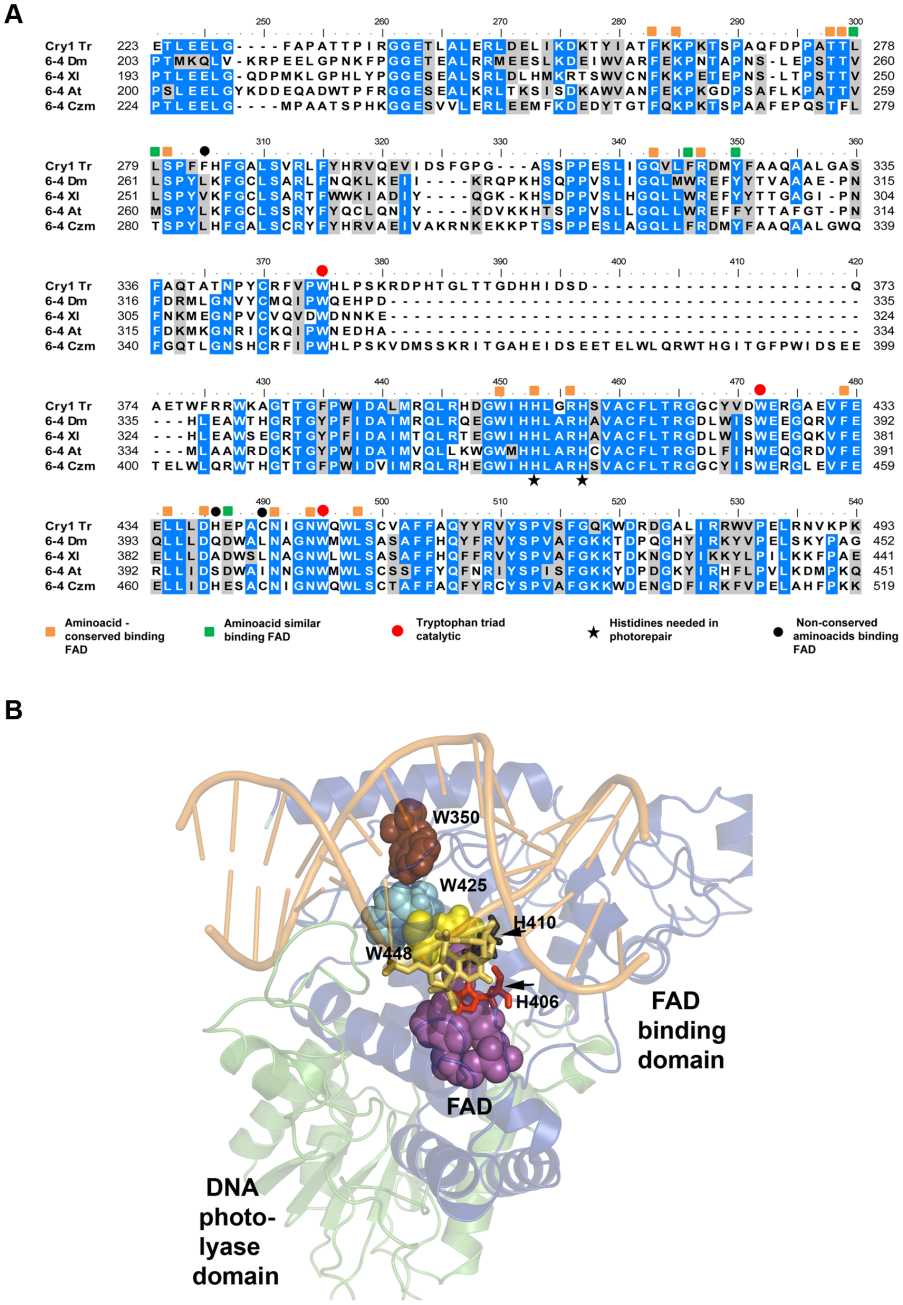
Comparative sequence analyses. A) Sequence alignment among 6–4 photolyases and Cry1. Conserved (white on blue) and similar (black on gray) aminoacids are high-lighted; red circles indicate the tryptophan triad; amino acids binding FAD are indicated by orange squares (conserved) and green squares (similar), as well as the histidines needed for photorepair (black stars). Black circles indicate the non-conserved amino acids in the FAD binding region. Cry1 *T. reesei* (Cry1 Tr), 6–4 photolyase *A. thaliana* (6–4 At), 6–4 photolyase *D. melanogaster* (6–4 Dm), 6–4 photolyase *X. leavis* (6–4 Xl) and Phl1 *C. zeae-maydis* (6–4 Czm). **B**) Homology model of Cry1. The DNA photolyase and FAD domains are represented as ribbon and colored in cyan and blue respectively. Catalytic histidines 406 and 410 are in a ball-stick representation, the tryptophan triad and FAD are represented as spheres. The non-conserved C-terminal extension of Cry1 was left from the model.

The *cry1* gene contains 1945 bp, including a 58 bp intron close to the 5' end of the coding region, resulting in a 1887 pb mature transcript encoding a predicted protein of 628 amino acids, with an estimated molecular weight of 71 kDa. We aligned the amino acid sequence of Cry1 (accession number, XP_006964927.1) using the Basic Local Alignment Search Tool (BLAST) to determine the existence of possible conserved domains. The BLAST sequence alignment allowed us to determine that the N-terminal region of Cry1 contains a conserved DNA photolyase domain (Pfam00875) between amino acid positions 6 and 173, and a FAD-binding domain (Pfam03441) in the amino acid positions 239 to 549, involved in binding a light harvesting cofactor; while the C-terminal domain (residues 550 to 628) does not show similarity to any known domain. We selected fifty-four reference sequences that represent the family members of cryptochromes/photolyases, including fungal protein sequences (see materials and methods). Reference sequences were retrieved from the National Center for Biotechnology Information protein database, and aligned for the construction of a phylogenetic tree. The classification of the subfamilies cryptochrome/6-4photolyase (blue), cryptochrome DASH (green) and CPD photolyases (red) and bacterial cryptochromes and photolyases (black) is shown in figure 1. The phylogenetic analysis of the cryptochrome/photolyase family across different species revealed that Cry1 is categorized as a cryptochrome/6-4 photolyase (shown in red, Fig.1). We then aligned the amino acid sequence of *T. reesei* Cry1 with three well characterized 6-4 photolyases; UVR3 of *A. thaliana*, PHR6-4 of *D. melanogaster*, Xl64PHR *of X. laevis*, and the Phl1 of *C. zeae-maydis* that is the only fungal 6-4 photolyase reported to date. The amino acid alignment depicts the conservation of the catalytic triad of tryptophan residues (Fig. 2A, red circles), the presence of two histidines needed to carry out photorepair (Fig. 2A, black stars) and twenty one amino acids, sixteen of which are identical and five similar, required for binding FAD of the twenty four reported in *A. thaliana* by Hitomi *et al.* [42] (Fig. 2A, squares). A structural model of Cry using as a template the crystal structure of a photolyase from *Drosophila melanogaster* in complex with 6-4 thymine dimer (PDB ID: 3CVU), depicts the possible conservation of the fold and places the catalytic amino acids and the tryptophan triad in a position to interact with the photolesion (Fig. 2B). These analyses place Cry1 within the cryptochrome/6-4 photolyase subfamily.

### *cry1* mRNA accumulates in response to blue-light in a *blr1* dependent manner and is modulated by Envoy

**Figure 3:**
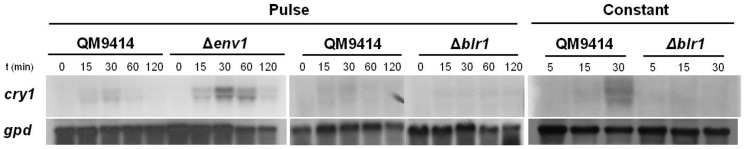
Expression of *cry1* in response to blue light. Analysis of the expression of *cry1* induced by a pulse of blue light (1200 µmol m^−2^) or upon exposure to constant light (fluence: 3.6 µmol m^−2^ s^−1^) for 72 h growth, as indicated. Transcript levels of the *cry1* gene were determined by Northern blot analysis of the parental strain QM9414, Δ*env1*, Δ*blr1* strains. The *gpd* gene was used as loading control in the different conditions.

As mentioned above changes in gene expression in response to light have been observed previously, both in *T. reesei* and *T. atroviride*. Including, in the latter, increased accumulation of the transcript of the cpd-photolyase encoding gene *phr1* [27], [4]. To determine whether *cry1* expression is affected by light, we analyzed its expression by Northern blot. We analyzed the expression of *cry1* after exposure to a pulse of blue light in the parental strain, and the Δ*blr1* and Δ*env1* mutants. No detectable levels of *cry1* transcript were observed in the parental strain when it was kept in the dark. Nevertheless, upon exposure to light *cry1* transcription could be detected, reaching its maximum level 30 min after exposure, and decreasing by 120 min (Fig. 3). In the *env1* mutant, transcripts of *cry1* accumulated to higher levels than in the parental strain (Fig. 3). No expression of *cry1* could be detected in the Δ*blr1* mutant strain, at any of the time points analyzed after light exposure (Fig. 3). Under constant exposure to blue light the results are similar to the treatment with a light pulse, showing maximum expression levels by 30 min in the parental strain and no detectable expression of *cry1* in the Δ*blr1* mutant (Fig. 3). Interestingly, two clearly distinct transcript bands were observed in the Northern analysis whenever expression of the gene could be detected. This observation suggests that *cry1* is subjected to differential splicing, although the relative abundance of the two transcripts didn't vary under the tested conditions. Our results suggest that *cry1* transcription is induced by light through Blr1, and repressed by Envoy.

### Cry1 is dispensable for growth

**Figure 4:**
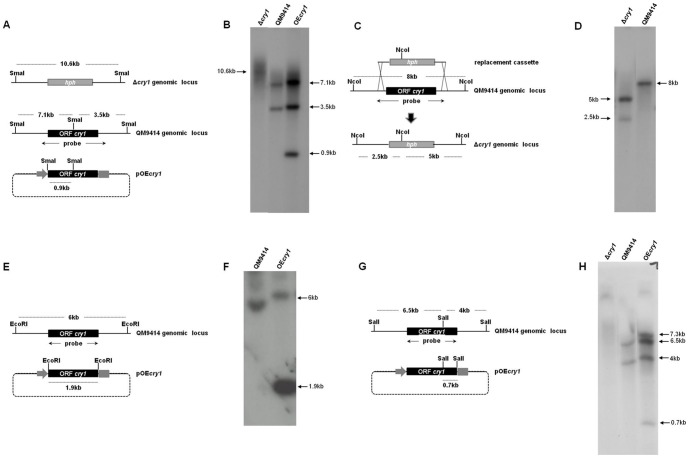
Molecular analysis of *cry1* mutant and overexpressing strains. Ten micrograms of genomic DNA were digested, separated on 1% agarose gel and hybridized with the probe indicated in the scheme. The position for each restriction enzyme and the sizes of the DNA fragments generated as indicated in each diagram. **A**) Schematic representation of Δ*cry1* genomic locus, parental genomic locus *cry1* and pOE*cry1*, digested with *Sma*I enzyme. **B**) Southern blot analysis of the parental strain (QM9414), overexpressing (OE*cry1*) and *cry1* mutant (Δ*cry1*). **C**) Schematic representation of replacement *cry1::hph*, digested with *Nco*I enzyme. **D**) Southern blot analysis of the parental strain (QM9414) and *cry1* mutant (Δ*cry1*). **E**) Schematic representation of parental genomic locus of *cry1* and the pOE*cry1*, digested with *EcoR*I enzyme. **F**) Southern blot analysis of the parental strain (QM9414) and overexpressing (OE*cry1*) strains. **G**) Schematic representation of pOE*cry1* and the parental genomic locus of *cry1*, digested with *Sal*I enzyme. **H**) Southern blot analysis of the parental strain (QM9414), overexpressing (OE*cry1*) and *cry1* mutant (Δ*cry1*) strains.

**Figure 5:**
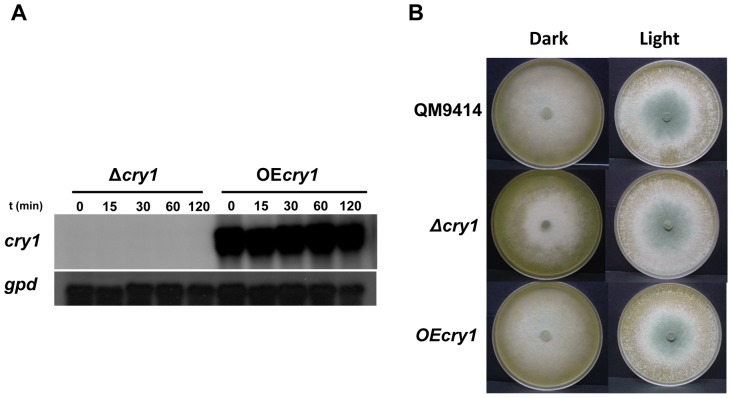
Analysis of the transformants for growth and expression in response to blue light. **A**) Growth of transformants on PDA under constant light and dark for 72 h, fluence: 3.6 µmol m^−2^ s^−1^. **B**) Analysis of the expression of *cry1* in transformants after exposure to a 5 min pulse of blue light (1200 µmol m^−2^). Transcript levels of the *cry1* gene were determined by Northern blot analysis and the *gpd* gene was used as loading control.

To elucidate the function of Cry1, we replaced the corresponding open reading frame by a hygromycin B-resistance cassette (*hph*). The DNA sequence of *cry1* was used to design oligonucleotides for its replacement using a double-joint PCR strategy. To demonstrate integration of the replacement cassette, we analyzed the parental strain, and the PCR-positive transformant (Δ*cry1*), by Southern blot. Genomic DNA from both strains was digested with the restriction enzyme *Sma*I and hybridized with a DNA fragment containing the promoter sequences, ORF and terminator of *cry1*, resulting in two bands (7.1 kb and 3.5 kb) in the parental strain and a single band (10.6 kb) in the *cry1* mutant, as expected (Fig. 4A and B). Additionally, the genomic DNA of the parental strain and *cry1* mutant was digested with *Nco*I and hybridized with a DNA fragment containing the promoter sequences, ORF and terminator of *cry1*, resulting in a band (8 kb) in the parental strain and two bands (5 kb and 2.5 kb) in the *cry1* mutant as expected (Fig. 4C and D). We also obtained transformants that should overexpress *cry1*, under the control of the *T. reesei pki* promoter. To demonstrate integration of the vector pOE*cry1*, we analyzed the parental strain, and the PCR-positive transformant (OE*cry1*), by Southern blot. Genomic DNA from both strains was digested with *EcoR*I and hybridized with a DNA fragment containing the ORF of *cry1*, resulting in a single band (6 kb) in the parental strain and two bands (6 kb and 1.9 kb) in the OE*cry1*, as expected (Fig. 4E and F). Similarly, when genomic DNA of the parental and overexpressing OE*cry1* strains was digested with *Sal*I and hybridized with a DNA fragment containing the *cry1* ORF, resulted in two bands (6.5 kb and 4 kb) for the endogenous copy of the gene in both strains, and the expected single band (0.7 kb) corresponding to the introduced copy of *cry1* in the OE*cry1*, an additional band (7.3 kb) was observed in the OE*cry1* strain, which is explained if integration of the vector occurred in the site of the *cry1* locus by homologous recombination (Fig. 4G and H). We analyzed the expression of *cry1* in the gene replacement mutant, and the overexpressing strain after a 5 min pulse of blue light (1200 µmol m^−2^) by Northern blot. No expression was detected in the Δ*cry1* mutant, while in the overexpressing strain very high constitutive levels of the *cry1* transcript were observed even in the dark, as expected (Fig. 5A), *cry1* expression of the parental strain QM9414 is shown in figure 3A (control). To analyze the behavior of both the *cry1* gene replacement mutants and the overexpressing strains, colonies of the Δ*cry1*, the OE*cry1*, and the parental strain were grown under continuous exposure to blue light or in the dark for 72 h at 28°C. The results show that neither the gene replacement mutant nor the overexpressing strain had any colony morphology alterations, and conidiated normally (Fig. 5B), it is important to mention that two independent gene replacement mutants and overexpressing strains were tested, and showed the same behavior. This may indicate that Cry1 has no participation in aspects related to growth at least under the tested conditions.

### Cry1 is a functional 6–4 photolyase

**Figure 6:**
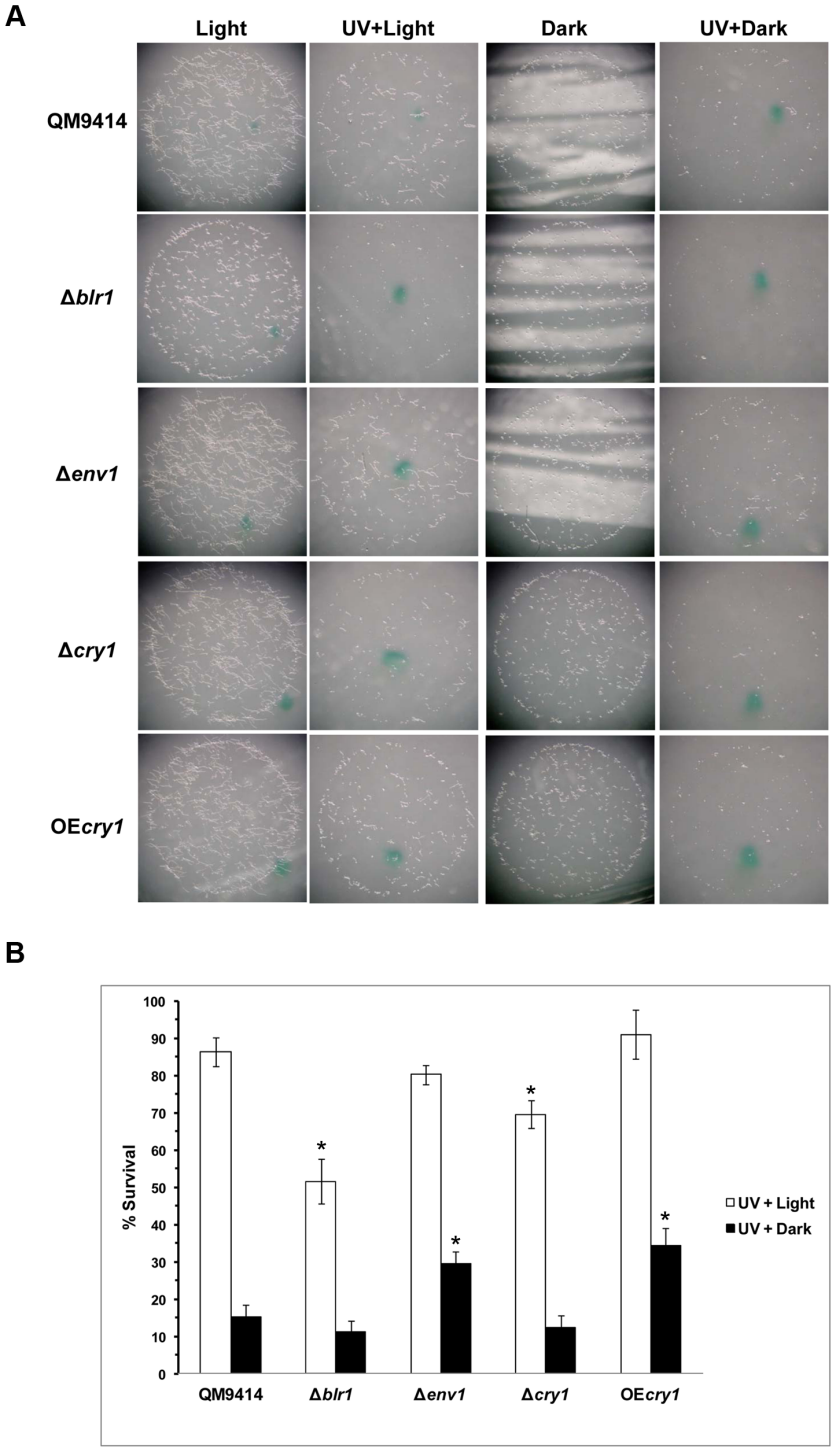
Photoreactivation assay *Trichoderma reesei*. **A**) Two hundred conidia of the strain indicated at the left of the figure were placed on PDA, and irradiated or not with UV light at 350 J m^−2^, then incubated at 28°C for 18 h in a chamber with white light or kept in the darkness, as indicated. The images were taken at a 20X amplification with a binocular microscope. **B**) Colonies of the experiment described in **A** were counted and the results plotted as percent survival for each condition in relation to the control non-irradiated with UV light. Bars indicate standard deviation from two independent experiments. The statistical analysis included one-way ANOVA with a significance level of p<0.05. An asterisk indicates that strains are significantly different from the QM9414 strain in each treatment.

To investigate if Cry1 could function as a 6–4 photolyase *in vivo* the photoreactivation activity of Cry1 was further analyzed in *T. reesei*. For this purpose, conidia from the parental, Δ*cry1*, OE*cry1*, Δ*blr1* and Δ*env1* strains were exposed to UV light (Fig. 6A). Subsequently, plates were exposed to light for photoreactivation or kept in the dark (control), and incubated in the dark for 48 h to determine the number of surviving colonies. The parental strain showed a great recovery in light (85%) compared to darkness (15%), in the Δ*blr1* mutant photoreactivation was 50%, about half of that observed in the parental strain, and repair in the dark was also slightly lower (10%). The Δ*env1* mutant showed a similar level (80%) of photorepair to that of the parental strain, but showed a significant increase in repair in the dark (30%). In the Δ*cry1* mutant a clear decrease in photoreactivation (70%) and no difference in repair in the dark (12%) were observed, as compared to the parental strain; two independent gene replacement mutants were analyzed and showed similar results. The *cry1* overexpressing strain showed a tendency to be more efficient in photorepair (average 90%) than the parental, but the difference was not statistically significant; two independent overexpressing strains were analyzed and showed similar results. These data indicate that Cry1 is involved in the process of photoreactivation in *T. reesei*. Interestingly, the overexpressing strain was clearly more tolerant to UV light exposure, as indicated by its survival rate (30%) when placed in the dark immediately after the UV-C treatment, a similar rate to that observed for the Δ*env1* mutant, which may indicate that Cry1 participates in light independent repair processes, or stimulating components of other repair systems such as NER [33], [43].

**Figure 7:**
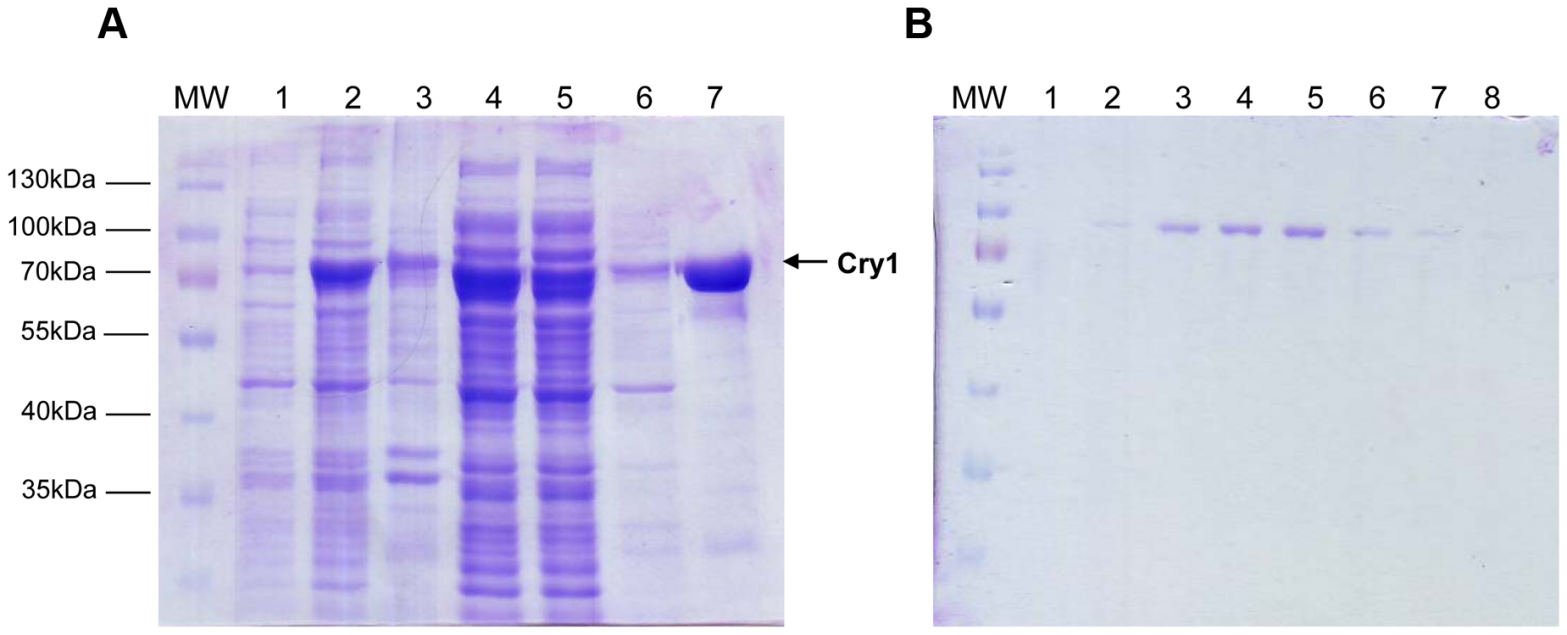
Heterologous expression and Purification of Cry1. **A**) Expression of Cry1 in *E.coli* SY2 (a DNA repair-defective strain) and purification using a HIS-Trap FF column. 1. Non induced; 2. Induced with 0.1 mM of IPTG; 3. Insoluble fraction; 4. Cell free extract; 5. Flow through a nickel column; 6. Fraction of unbound proteins; 7. Fraction eluted with Imidazole 500 mM. **B**) Purification of Cry1 using a monoQ anion exchange column eluting with a NaCl gradient. 1. Flow through a monoQ column. 2–8. Fractions eluted with NaCl 50, 100, 200, 300, 400, 500, and 600 mM, respectively. MW. Molecular weight marker.

**Figure 8:**
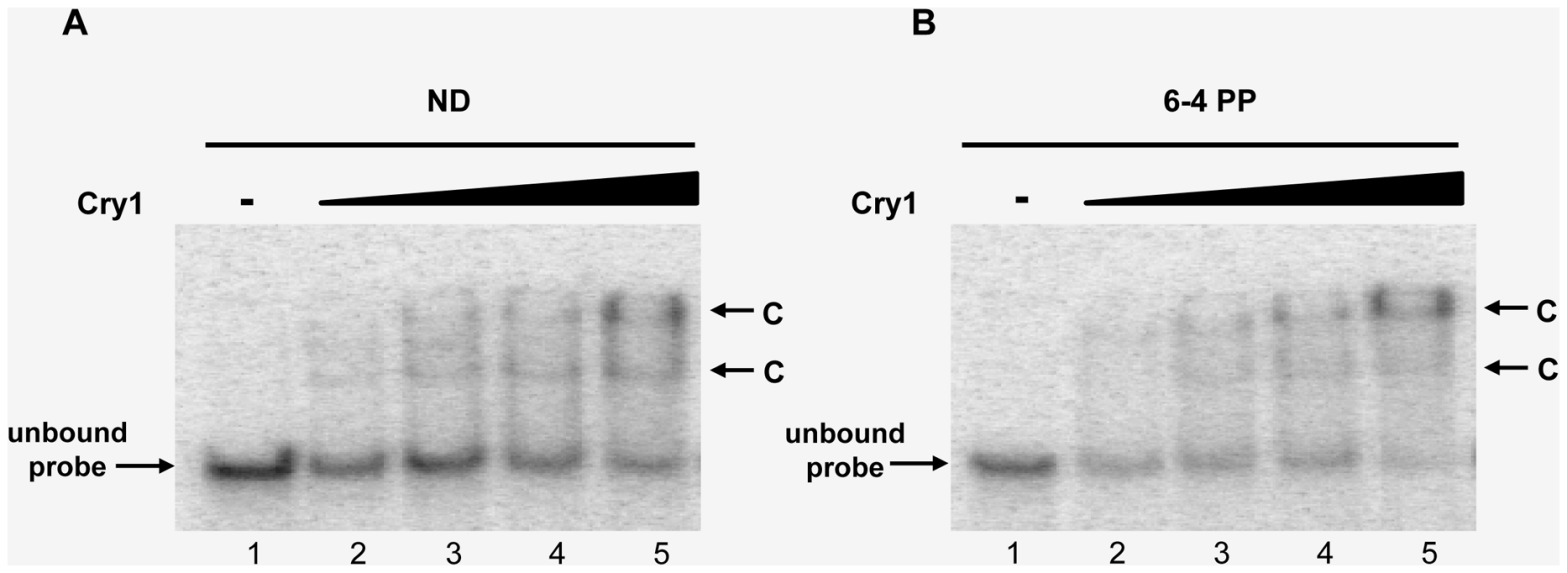
Binding of Cry1 to DNA. EMSA Assay using: non-damage oligomers **A**) and 6–4 PP oligomers **B**). An arrow preceded by a C indicates the migration of oligomer-Cry1 complexes. 1 nM oligo-labelled, Cry1: 0, 4.23, 8.45, 12.68 and 25.35 µM respectively in lines 1–5.

**Figure 9:**
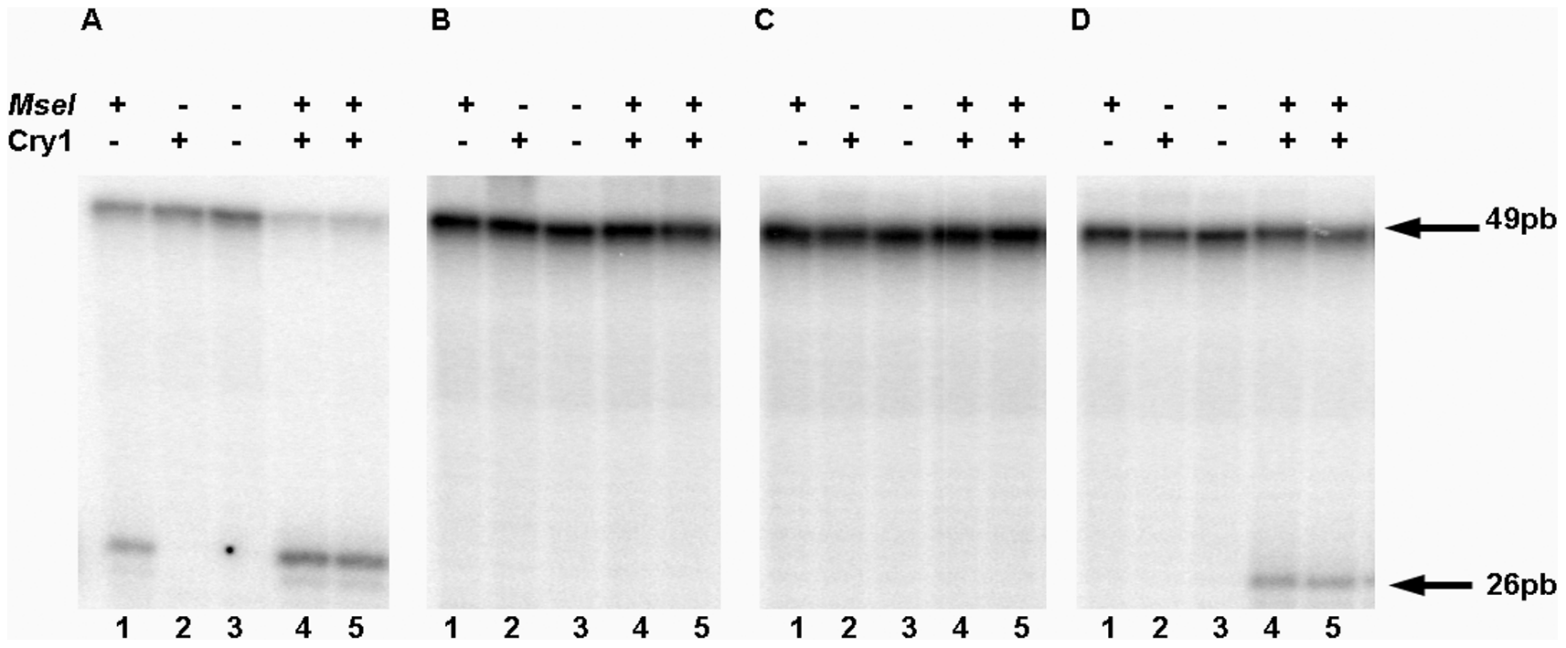
*In vitro* Photorepair Assay. Cry1 (7 µM) was incubated with 10 nM labeled oligonucleotides for 20 min under constant white light for photoactivation. After photoreactivation, the DNA was digested with *Mse*I and separated on a 10% polyacrylamide gel. The digested product with *Mse*I indicates photorepair of the substrate: **A**) Undamaged oligomer. **B**) CPD oligomer. **C**) Dewar oligomer. **D**) 6–4 PP oligomer. (+) Indicates presence and (−) absence of the enzyme indicated at the left.

The Cry1 protein was expressed in *E. coli* SY2 (recA^−^, phr^−^, uvrA^−^) and purified to homogeneity by affinity and ion exchange chromatography. After ion exchange the heterologous Cry1 protein is observed as a single protein band by SDS-PAGE (Fig. 7). In order to biochemically characterize the recombinant expressed Cry1, we tested the ability of the protein to bind DNA using an EMSA assay. Gel shift electrophoresis was carried out with oligonucleotides without damage, and with 6-4PP damage. In both cases DNA-protein complexes were observed. The formation of the complex increased with increasing amounts of protein in a similar proportion for damaged and undamaged DNA substrates. Thus, Cry1 binds DNA independently of the presence of 6-4PP (Fig. 8). To determine if Cry1 has 6–4 photolyase activity, photorepair activity assays were performed *in vitro*. We tested the ability of Cry1 to repair DNA damage caused by UV light *in vitro*. We performed an assay that determines the ability of the enzyme to repair DNA damage using an oligonucleotide that contains an *Mse*I restriction site in which both thymines are UV damaged. Thus, if repair occurs the damaged thymines restore a functional *Mse*I recognition site and *Mse*I cleavage would indicate repair activity. We observed, absence of DNA repair activity when Cry1 was applied to oligonucleotides bearing CPD damage or DEWAR (Figs. 9B and C). In contrast, our results clearly indicate that Cry1 has 6–4 photolyase repair activity, restoring the *MseI* site, which can then be cleaved into the two expected 23 bp and 26 bp fragments. (Fig. 9D)

## Discussion

Cryptochromes/photolyases have recently been characterized to some extent in fungi such as *S. sclerotiorum*, *C. zeae-maydis*, *N. crassa*, and *F. fujikuroi* [30], [33], [31], [32] but their function is still not completely understood. Here we describe the cloning and characterization of Cry1, which should be classified as part of the cryptochrome/photolyase family. In fact, our phylogenetic data indicate that it belongs to the cryptochrome/6-4 photolyase subfamily, as previously reported [8]. The BLAST alignments show that there are several fungal hypothetical proteins annotated in the database with a very high identity to the *T. reesei* Cry1. Cry1 shows 37% identity with the 6–4 photolyase (UVR3) of *A. thaliana*, 41% identity with the 6–4 photolyase (PHR6-4) from *D. melanogaster* and 61% with PHL1 of *C. zeae-maydis* [33]. Cry1 presents the characteristic 6–4 photolyase conserved domain and the residues required for DNA repair, and a 79 amino acids C-terminal region that has no similarity to any domain in the database. However, a putative nuclear localization signal was found in the C-terminal region, as it has been found in animal cryptochromes that regulate the circadian clock [18], [44]. Unfortunately, in fungi there are no reports on the function of this C-terminal region in cryptochromes.

The *T. reesei* Cry1 protein sequence contains amino acids that carry out important functions such as the FAD binding domain that has been described in the 6–4 photolyases of *A. thaliana*, *X. laevis*, *D. melanogaster*, and *D. salina* [41], [45], [42], [46], [47], [48], as well as a pair of histidines (His 406 and His 410) also found in the *X. laevis* (Xl64PHR), and *A. thaliana* (UVR3) 6–4 photolyases, necessary to carry out photoreactivation, and lysine 263 also proven to participate in photoreactivation in *D. salina* (Ds64PHR), but in a pH dependent manner, and the catalytic tryptophan triad (Trp 350, Trp 425 and Trp 448) involved in the electron transfer during photoreactivation in *A. thaliana*.

In previous reports where light regulated gene expression was analyzed, it was found that early response genes, reach a maximum within 30 min and their expression decreases by 120 min after a light pulse [4], [49], [36]. Consistently, *cry1* mRNA accumulates in response to blue light, reaching its maximum expression by 30 min, and therefore should be considered an early response gene. The Blr proteins are known to regulate the majority of light responsive genes, a high percentage of the promoter regions of those genes contain GATA type consensus sequences called LREs (light response elements). LREs have been proposed to be the binding target of the Blr proteins [4]. In agreement with its transcriptional control by Blr1, the *cry1* promoter also contains these potential light response elements. The elevated *cry1* mRNA levels observed in our experiments in the Δ*env1* mutant supports the proposed role of Envoy as a negative regulator of light perception in *Trichoderma*.

Members of the cryptochrome/photolyase family have been reported to play different roles in fungal biology, including photoreactivation, control of gene expression, secondary metabolism, and development [28], [29], [30], [33], [31], [32]. However, most of those reports refer to CPD photolyase or DASH type cryptochromes, except for Phl1 of *Cercospora*, which has 6–4 photolyase activity and participates in regulation of gene expression [33]. The close similarity of the *T. reesei* Cry1 to Phl1 of *C. zeae-maydis* suggested its possible involvement in development and metabolism [33]. Nevertheless, no morphological defects were observed in either the gene replacement mutants or the overexpressing strains, neither in the light nor in the dark. Thus, Cry1 doesn't appear to play a major role in the physiology of *T. reesei*, except in the repair of DNA damage, at least under the conditions tested in this work. When we analyzed the capacity of photoreactivation of the mutant strains compared to the parental strain, a small but significant decrease was observed, providing evidence of the involvement of Cry1 in photoreactivation. This small reduction in the photoreactivation capacity of the fungus is in agreement with the fact that 6-4PP represent only 10–25% of all UV-damaged DNA [15]. This result contrasts with those obtained in *C. zeae-maydis* where *phl1* mutants showed a drastic reduction in photoreactivation. In the case of *Cercospora*, the authors suggested that that the drastic reduction in photoreactivation was due to the regulation of the CPD photolyase activity by Phl1 [33]. In contrast, in our case, expression of *phr1* (a CPD photolyase) does not appear to depend on Cry1, since most of the photoreactivation capacity is maintained in the *T. reesei* Δ*cry1* mutants. Moreover in the Δ*blr1* mutant we observed a dramatic decrease in photoreactivation, which can be explained by the light induced expression of both the 6–4 photolyase (Cry1) and the CPD photolyase (Phr1), which is under the control of Blr1 [28], [3]. Although the Δ*env1* mutant and the overexpressing strain OE*cry1* accumulated much more *cry1* mRNA, they did not show significant difference in photorepair, as compared to the parental strain. Interestingly, colonies of these strains showed a significant increase in their recovery after exposure to UV than the parental strain in the dark. These observations suggest that Cry1 may be stimulating other systems involved in DNA repair, such as the nucleotide excision repair system (NER) [33], [43]. Thus, implying a similar evolution in the control of DNA repair systems to that reported in *C. zeae-maydis* where Phl1 regulates expression of genes that are part of the NER system [33]. Alternatively, Cry1 may interact with proteins that activate other repair systems. Most 6–4 photolyases characterized so far show high specific binding to damaged DNA containing 6-4PP [50], [51], [41], [52], [53], except for UVR3 from *A. thaliana*, which has clearly detectable binding to undamaged DNA [53], [54]. Interestingly, we observed only a very slight difference in the binding affinity of Cry1 to 6-4PP damaged and undamaged DNA. This could simply be due to non-optimal conditions in our binding assay. An attractive alternative, however, would be that Cry1 has lost specificity to bind damaged DNA, and has gained regulatory functions, which could require binding to undamaged DNA.

Not all fungi have in their genome genes coding for proteins with 6–4 photolyase activity, such is the case of *N. crassa* which is one of the most studied ones in the field of photobiology, and which, like *Trichoderma*, belongs to the the Sordariomycetes. In this sense, it was previously believed that 6–4 photolyase activity was unique to some eukaryotes. However 6–4 photolyase activity has recently been found even in bacteria [13], suggesting the existence of selective forces acting on particular organisms. It is thus, of major interest for future work to analyze the evolution of this type of DNA repair proteins through different taxa. Moreover, an interesting question is whether this group of fungal proteins have only 6–4 photolyase repair activity or if they have regulatory functions as described in algae [52], [53], and the fungus *Cercospora zeae-maydis* [33].
